# A Rare Case of Urinary Bladder Leiomyoma Invading Urachal Remnant Managed With Robotic Partial Cystectomy

**DOI:** 10.7759/cureus.64222

**Published:** 2024-07-10

**Authors:** Mohammed Elsheikh, Jon Oxley, Farukh Qureshi, Mark Thornton

**Affiliations:** 1 Urology, Royal Bournemouth Hospital, University Hospital Dorset, Bournemouth, GBR; 2 Pathology, Southmead Hospital, North Bristol National Health Service (NHS) Trust, Bristol, GBR; 3 Urology, Southmead Hospital, North Bristol National Health Service (NHS) Trust, Bristol, GBR; 4 Radiology, Southmead Hospital, North Bristol National Health Service (NHS) Trust, Bristol, GBR

**Keywords:** bladder ca, partial cystectomy, transurethral resection of bladder tumor, urinary bladder leiomyoma, kidney-adrenal-bladder-prostate-renal transplant-ct-mri-nuclear medicine, bladder tumours, bladder mri, bladder imaging, bladder leiomyoma, bladder

## Abstract

Leiomyoma is a rare benign tumour of the urinary bladder. Typically, bladder leiomyomas are treated with transurethral resection, which yields favourable results. We present a clinical case of a 29-year-old man with a symptomatic bladder tumour, initially diagnosed on flexible cystoscopy and CT scan. Subsequent transurethral resection and MRI scan confirmed a transmural bladder leiomyoma invading the urachal remnant. The patient was subsequently treated with robotic partial cystectomy. The presentation and management, including imaging and histopathology results, are discussed with a brief review of the literature.

## Introduction

Leiomyoma of the urinary bladder is a rare benign mesenchymal tumour that accounts for about 0.4% of all bladder tumours [1]. Most patients may present with urinary frequency or voiding urinary symptoms such as hesitancy and dribbling. Imaging techniques such as ultrasonography, computed tomography, and magnetic resonance imaging can be useful; however, a definitive diagnosis is established through histopathological examination of the tumour. The primary treatment involves surgical excision, typically via transurethral resection (TURBT). In certain cases, open, laparoscopic, or robot-assisted surgical approaches may be considered. We present a case of bladder leiomyoma managed at our institution with a review of other cases reported in the literature.

## Case presentation

**Figure 1:**
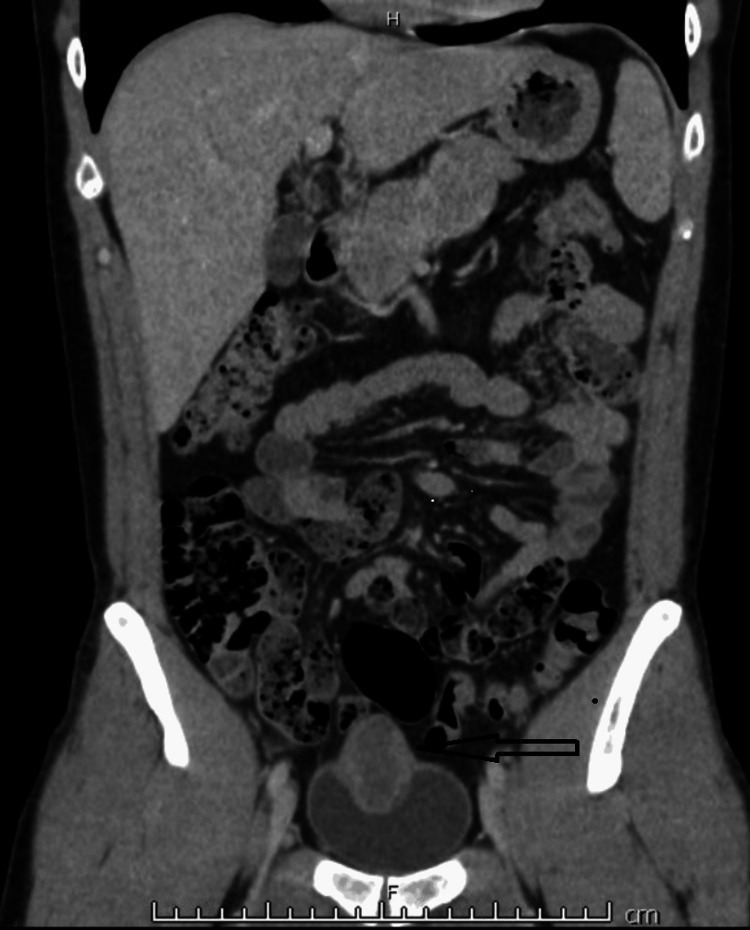
Coronal section of CT abdomen/pelvis demonstrating the apical bladder mass

A 29-year-old man presented via his general practitioner with visible haematuria and increasing dysuria with no evidence of infection on both midstream urine culture and sexually transmitted infection testing. Clinical examination was unremarkable, with no palpable abdominal masses, normal external genitalia, and a benign-feeling, small prostate gland. Flexible cystoscopy revealed a 4 cm solid mass at the dome of the bladder. A CT scan (Figure 1) revealed a well-defined 46 x 33 cm lobulated anterior bladder mass which appeared to extend through the anterior bladder wall involving the urachal remnant with a radiological staging of T3 N0 M0. Transurethral resection (TURBT) was carried out and histopathology was consistent with infarcted leiomyoma of the bladder.

**Figure 2:**
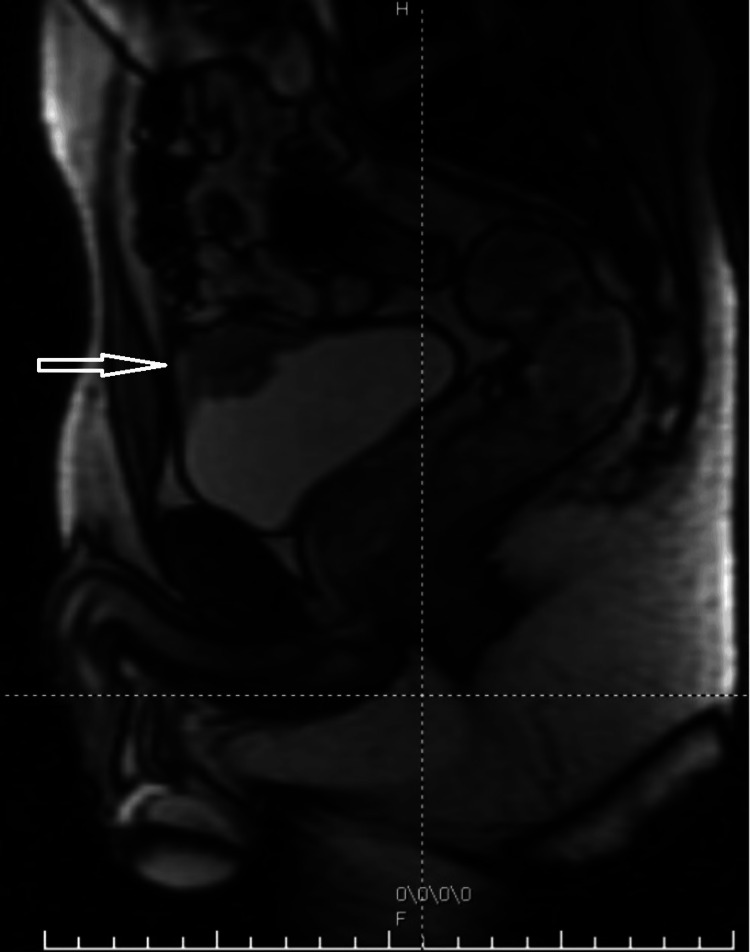
MRI pelvis (sagittal view) The image demonstrates an apical bladder tumour involving a urachal remnant.

Postoperative MRI pelvis (Figure 2) was performed approximately eight weeks after TURBT and confirmed tumour de-bulking with a persistent 3 cm transmural tumour involving the lower part of the urachus. The patient remained symptomatic and following a multidisciplinary team review, robotic-assisted partial cystectomy with excision of the urachal remnant was performed.

**Figure 3:**
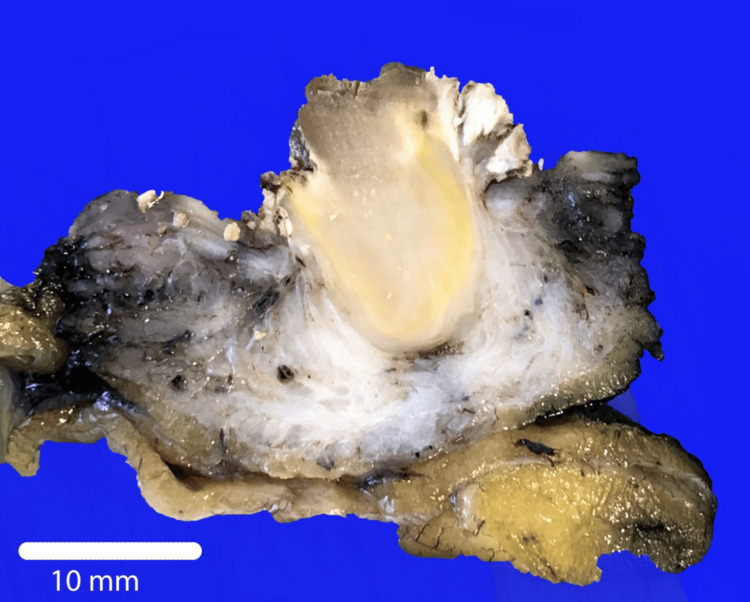
Macroscopic image of the tumour

**Figure 4:**
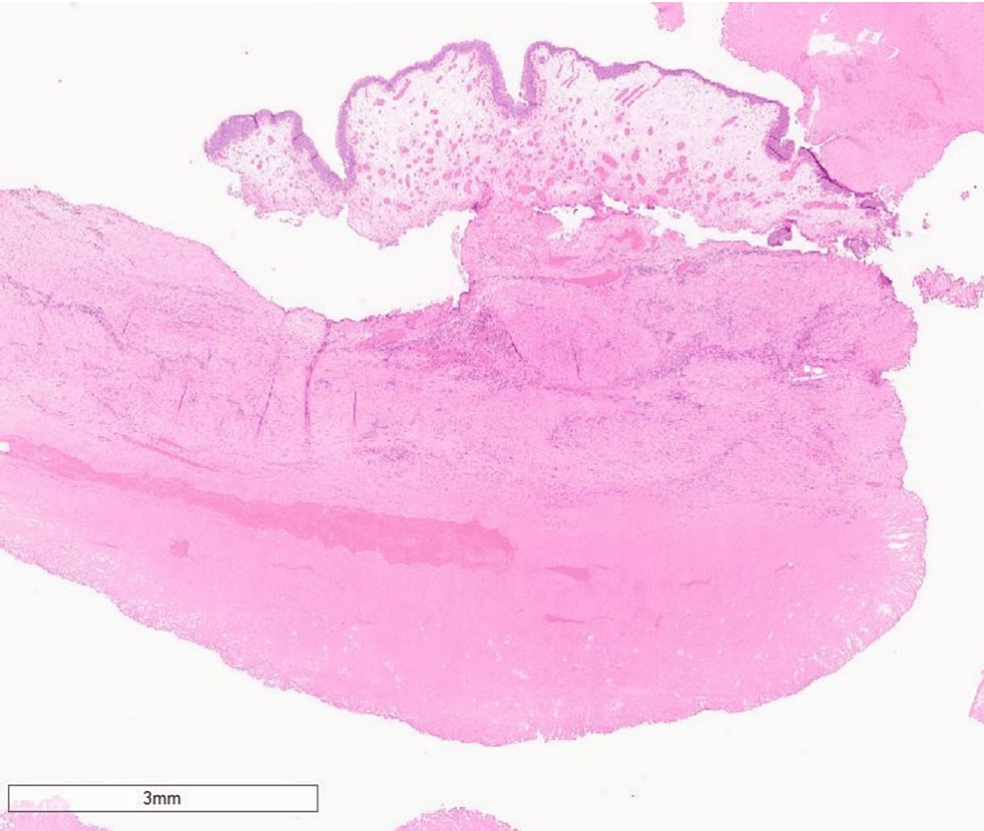
Low-power histopathology image The image is showing lamina propria oedema and deeper muscle necrosis.

**Figure 5:**
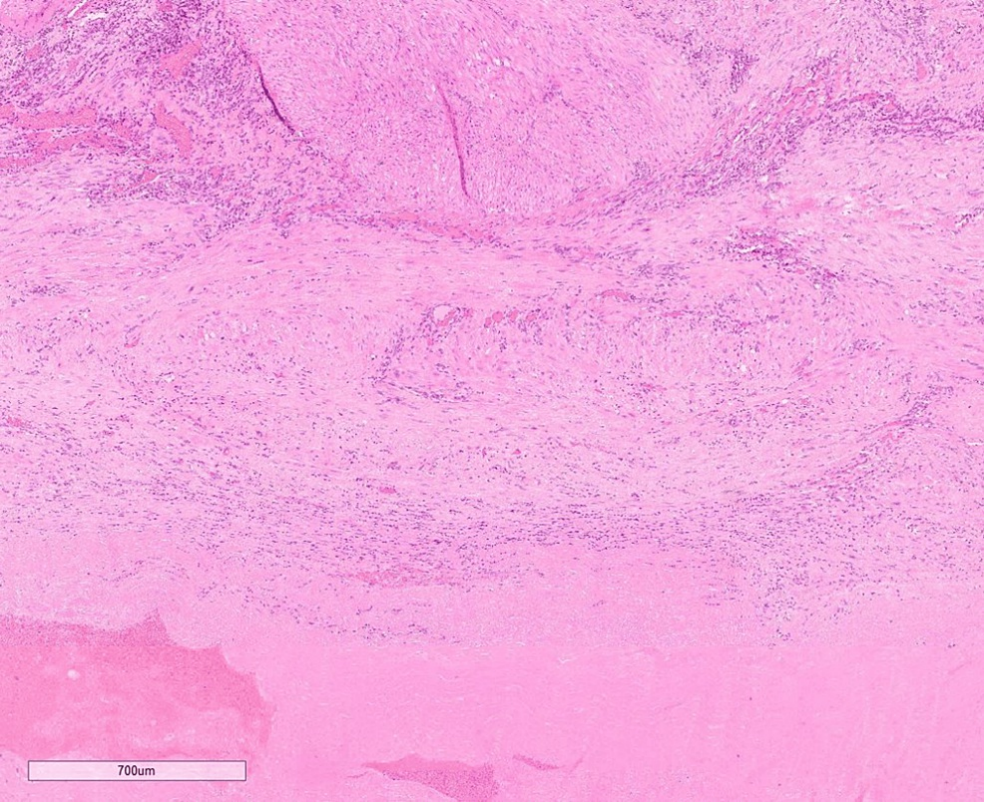
High-power histopathology image The image is showing the muscle bundles with foci of necrosis.

The patient made a good recovery and was discharged to their home two days following the surgery. Histopathology validated the diagnosis of infarcted bladder leiomyoma, with no signs of urachal cancer (Figures 3-5). Our plan is to monitor him based on his symptoms following the complete excision of the tumour.

## Discussion

Leiomyomas of the urinary bladder are uncommon neoplasms, with only about 250 cases reported previously [2]. Approximately 75% of the patients are young to middle-aged [1]. The etiology of bladder leiomyomas remains unclear. Some research suggests that it may be driven by oestrogen as oestrogen receptors have been identified in some tumours [3]. An association between pulmonary lymphangioleiomyomatosis and subsequent bladder leiomyoma presentation has been reported [4]. Although it may be asymptomatic in 20% of the cases, the majority present with voiding or storage urinary symptoms and it may present with pelvic pain, dysuria, haematuria or rarely dyspareunia in females [5]. Ultrasound is commonly employed as the primary imaging technique; however, CT and MRI can be instrumental in determining the tumour's location, invasion depth, and size and in evaluating the entire urinary tract [6]. On MRI, bladder leiomyomas exhibit low intensity on both T1- and T2-weighted sequences and have a smooth perimeter, resembling a uterine leiomyoma. Following gadolinium injection, various enhancement patterns may be observed; some leiomyomas may show homogeneous enhancement, while others may not. However, imaging techniques alone cannot rule out malignancy, thus a tissue sample is invariably required [7]. Cystoscopy often reveals a distinctive bladder mass covered by smooth, regular mucosa. Some tumours have been reported to reach sizes up to 25 cm [7].

Leiomyomas of the urinary bladder exhibit histopathological characteristics similar to those of uterine leiomyomas, such as round nodules, a grey-white colour, and a whorled pattern of smooth muscle fibres arranged in small bundles. These are interspersed with varying amounts of fibrous connective tissue and typically show fewer than two mitotic figures per high-power field [8]. The treatment of bladder leiomyoma is contingent upon its size, location, and the patient's symptoms. In cases where the patient is asymptomatic and a biopsy has confirmed leiomyoma, it may be reasonable to monitor patient's symptoms and to schedule follow-up imaging at a future date.. In patients with symptoms from small bladder leiomyomas, transurethral resection (TUR) can be safely conducted. Documentation suggests that re-resection may be necessary in 18% of cases, potentially due to incomplete initial resection [9]. Intramural, extravesical, and large leiomyomas are optimally managed with partial cystectomy or enucleation. Laparoscopic, trans-vesical, and robotic surgical techniques can be employed effectively for this purpose [10-12]. The overall prognosis for bladder leiomyomas is favourable, and recurrence rates are low after surgical resection [6].

## Conclusions

Our case report highlights the challenges in managing atypical bladder tumours, particularly in symptomatic younger patients. In this instance, the patient underwent a partial cystectomy for treatment of symptomatic transmural leiomyoma as complete transurethral resection was unfeasible. The use of robotic techniques contributed to a faster recovery and reduced the length of hospital stay. We suggest following the patients who had complete resection or excision of bladder leiomyomas based on their symptoms, as no clear guidelines exist regarding follow-up of these cases. Continued research is crucial to establish the best treatment and monitoring strategies for patients with symptomatic bladder leiomyomas.
